# Sample size estimation for the averted events ratio

**DOI:** 10.1177/17407745251377435

**Published:** 2025-10-23

**Authors:** David T Dunn, Oliver T Stirrup, David V Glidden

**Affiliations:** 1 MRC Clinical Trials Unit, University College London, London, UK; 2 Institute for Global Health, University College London, London, UK; 3 Department of Epidemiology and Biostatistics, University of California San Francisco, San Francisco, CA, USA

**Keywords:** Active-control trial, non-inferiority, preservation-of-effect, averted events, 95-95 method, estimand, sample size

## Abstract

**Background:**

The averted events ratio (AER) is a recently developed estimand for non-inferiority active-control prevention trials with a time-to-event outcome. In contrast to the traditional rate ratio or rate difference, the AER is based on the number of events *averted* by each of the two treatments rather than the observed events. The AER requires an assumption about either the background event rate (the counterfactual placebo incidence) or the counterfactual effectiveness of the control treatment. We develop and present sample size formulae for trials in which the AER is defined as the primary estimand, and draw comparisons with the conventional 95-95 method based on the rate ratio.

**Methods:**

We express sample size in terms of the expected number of events and required person-years follow-up in the control and experimental arms. Sample size formulae were based on Wald confidence intervals on a logarithmic scale, assuming the active and control treatments to be equally effective. Using the AER, sample size depends on whether the analysis will be based on the counterfactual placebo incidence or the counterfactual treatment effectiveness. For both approaches, and the 95-95 method, sample size is a function of the background event rate, the effectiveness of the control treatment, the preservation-of-effect size (non-inferiority margin), the confidence limit for inferring non-inferiority, and the desired statistical power to demonstrate non-inferiority.

**Results:**

The smallest sample size is obtained using the AER based on the counterfactual placebo incidence. The advantage is greater the higher the value of the control treatment effectiveness. For example, compared with the 95-95 method, it allows between a 2.6-fold and 4.0-fold reduction in sample size for 50% treatment effectiveness (depending of the non-inferiority margin), and between a 7.7-fold and 11.9-fold reduction for 80% treatment effectiveness. The AER based on the control treatment effectiveness is less efficient but still requires smaller sample sizes than the 95-95 method: between a 1.5-fold and 2.9-fold reduction for 50% treatment effectiveness, and between a 2.3-fold and 6.4-fold reduction for 80% treatment effectiveness. Sample size is highly sensitive to the non-inferiority margin: increasing the preservation-of-effect size from 50% to 60% implies a 1.84-fold increase in the sample size; from 60% to 70%, an increase of 2.15-fold; and from 70% to 80%, an increase of 2.55-fold.

**Conclusion:**

As well as having important advantages of interpretation, using the AER as the primary estimand in active-control non-inferiority trials permits smaller and more cost-effective studies. Ideally, the AER should be derived via the counterfactual placebo incidence when this is practicable.

## Introduction

Active-control trials, where an experimental treatment is compared with a standard treatment, are performed when the inclusion of a placebo control group is considered unethical.^1^ They are often conducted within a non-inferiority framework, where a critically important design decision is the specification of the non-inferiority margin.^2,3^ One approach to this problem is to attempt to demonstrate that the experimental treatment preserves a minimum fraction (typically 50%) of the effect of the standard, conventional treatment (preservation-of-effect criterion).^4–7^ The 95-95 method is a specific application of this approach for time-to-event outcomes.^3,8,9^ The name of the method derives from the use of (a) the 95% confidence interval from a meta-analysis of historical trials comparing the control treatment versus placebo to derive a conservatively low estimate for the intrinsic effect of the control treatment and (b) the 95% confidence interval from the active-control trial to derive a conservatively low estimate for the effect of the experimental treatment relative to the standard treatment. Although the 95% values are arbitrary, they have largely become a *de facto* standard.

With the 95-95 method, inference is performed on a log rate ratio (or log hazard ratio) scale.^8,9^ However, we have pointed out that this can lead to major problems of interpretation.^10–12^ First, and most crucially, the method may fail to formally demonstrate non-inferiority even when the experimental treatment is demonstrably highly effective.^11^ Second, inference can be highly unstable, in that a slight re-distribution of endpoints between the two treatment groups can reverse the conclusion regarding non-inferiority.^11^ Third, it paradoxically gives more precise inference in less adherent populations than in highly adherent populations.^10^ Finally, the method is predicated on significance testing and lacks an interpretable estimand.^4^

These problems can be avoided by using an estimand we have developed, the averted events ratio (AER), which essentially considers inference on a rate difference scale.^10,12^ With this estimand, the experimental and standard treatments are compared in terms of *averted* events rather than observed events. The AER has a clinically appealing preservation-of-effect interpretation: the proportion of events prevented by using the experimental treatment that would otherwise have been prevented by the standard treatment.^10^

We have exemplified the AER in the field of HIV pre-exposure prophylaxis drugs taken by HIV-negative individuals to prevent the acquisition of infection.^13^ Several active-control, non-inferiority trials have been conducted using the licenced, two-drug combination tenofovir disoproxil fumarate and emtricitabine (TDF-FTC) as the control arm; all were designed and analysed using the 95-95 method.^14,15^ TDF-FTC is highly efficacious and reduces the risk of HIV acquisition by ~95% if taken as prescribed.^16^ These trials were consequently very large (>10,000 person-years follow-up (PYFU)) in order to generate the required number of endpoints. However, we have previously shown that much tighter inference on non-inferiority can be achieved if the analysis is performed via the AER, suggesting that smaller, more affordable, trials may be possible.^11,17^ In this article, we describe how to determine the sample size based on the AER and assess the reduction in sample size compared with the 95-95 method. We hope that this may stimulate the use of the AER, which, despite its broad applicability,^12^ has, to date, been only slowly adopted.

## Methods

### Definition of the AER

We conceptualise that the active-control trial includes a hypothetical third arm that receives no intervention (called the counterfactual placebo group), in addition to the experimental and control arms. The (unobserved) incidence rate in the counterfactual placebo group can also be thought of as the background incidence in the study population. Denote the counterfactual placebo, control, and experimental arms by the subscripts P, C, and E, respectively. Let λ (subscripted by P, C, or E) represent the relevant incidence rate. The AER is defined as

(1) $$\Psi_{\lambda }=\;\frac{\lambda_{P}-\;\lambda_{E}}{\lambda_{P}-\;\lambda_{C}}$$

Alternatively, the AER can be expressed in terms of the counterfactual effectiveness of the control treatment relative to placebo ( $\theta_{\mathrm{CP}}=1-\lambda_{C}/\lambda_{P\;})$ :

(2) $$\begin{array}{c} \Psi_{\theta } \end{array}=\;\frac{1-\lambda_{E}/\lambda_{C}\left(1-\theta_{\mathrm{CP}}\right)}{\theta_{\mathrm{CP}}}\;$$

We note that this formulation involves the standard rate ratio of the observed event rates. However, the transformation converts this into the ratio of the averted event rates.^18^

As will be shown in the next section, the required sample size depends on whether formulation (1) or (2) is used for the AER.

### Sample size for the AER

For simplicity, we assume that we observe equal PYFU, denoted by F, in the control and experimental arms. Let X_C_ and X_E_ be random variables denoting the number of observed events, where $X_{C}\sim \mathrm{Poi}\left(F\;\lambda_{C}\right)$ and $\;X_{E}\sim \mathrm{Poi}\left(F\;\lambda_{E}\right)$ . Non-inferiority is demonstrated if the lower (1 - α) confidence limit for the AER exceeds the pre-specified non-inferiority margin, Δ, that is, the smallest preservation-of-effect fraction that we wish to demonstrate.

Let β denote the probability of declaring non-inferiority (power), and define $Z\left(\alpha ,\beta \right)=\;{\left[\Phi^{-1}\left(1-\alpha \right)+\;\Phi^{-1}\left(\beta \right)\right]}^{2}$ .

We assume throughout that the experimental and control treatments are equally effective ( $\theta_{\mathrm{EP}}=\theta_{\mathrm{CP}}$ ). When estimating the AER via the counterfactual placebo incidence (equation (1)), the required expected number of events in each of the active treatment arms is (see Appendix in the Supplementary Material for derivation):

(3) $$\begin{array}{c} N_{\Psi_{\lambda }}=\;2\;Z\left(\alpha ,\beta \right)\;{\left[\frac{\left(1-\theta_{\mathrm{CP}}\right)}{\theta_{\mathrm{CP}}\;\log\Delta }\right]}^{2}\; \end{array}$$

Similarly, when estimating the AER via the counterfactual treatment effectiveness (equation (2)), the required expected number of events in each of the active treatment arms is:

(4) $$\begin{array}{c} N_{\Psi_{\theta }}=\;\frac{2\;Z\left(\alpha ,\beta \right)}{{\left[\log\left(1-\Delta \;\theta_{\mathrm{CP}}\right)-\log\left(1-\theta_{\mathrm{CP}}\right)\;\right]}^{2}} \end{array}$$

The ratio of the number of events required when using $\Psi_{\theta }$ rather than $\Psi_{\lambda }$ is

(5) $$\begin{array}{c} N_{\Psi_{\theta }}/N_{\Psi_{\lambda }}=\;{\left[\frac{\theta_{\mathrm{CP}}\;\log\Delta }{\left(1-\theta_{\mathrm{CP}}\right)\;\left[\log\left(1-\Delta \;\theta_{\mathrm{CP}}\right)-\;\log\left(1-\theta_{\mathrm{CP}}\right)\right]}\right]}^{2} \end{array}$$

### Sample size for the 95-95 method

With the 95-95 method, effect preservation is measured by $\phi =\beta_{\mathrm{PE}}/\beta_{\mathrm{PC}}$ , where $\beta_{\mathrm{PE}}=\log\left(\lambda_{P}/\lambda_{E}\right)$ and $\beta_{\mathrm{PC}}=\log\left(\lambda_{P}/\lambda_{C}\right)$ .^8,9^ (Note that $\beta_{\mathrm{PC}}=\log\;\left[1/\left(1-\theta_{\mathrm{CP}}\right)\right].$ ) Although mathematically convenient, in contrast to the AER, ϕ has no direct clinical interpretation. Analogous to $\Psi_{\theta }$ , the estimation of ϕ requires an assumption about the counterfactual effectiveness of the control treatment that would have been observed in the trial. As described in the Introduction, this is usually imputed from a meta-analysis of previous placebo-controlled trials (the so-called ‘constancy’ assumption).^7–9^ The required number of events per active arm with the 95-95 method is:

(6) $$\begin{array}{c} N_{95-95}=\;\frac{2\;Z\left(\alpha ,\beta \right)}{{\left[\log\left(1-\theta_{\mathrm{CP}}\right)\left(1-\Delta \right)\right]}^{2}} \end{array}$$

The ratio of the number of events when using the 95-95 method rather than $\Psi_{\lambda }$ is:

(7) $$\begin{array}{c} N_{95-95}/N_{\Psi_{\lambda }}=\;{\left[\frac{\theta_{\mathrm{CP}}\;\log\Delta }{\left(1-\theta_{\mathrm{CP}}\right)\;\log\left(1-\theta_{\mathrm{CP}}\right)\left(1-\Delta \right)\;}\right]}^{2} \end{array}$$

Alternatively, when the comparator is $\Psi_{\theta }$ , the ratio is:

(8) $$\begin{array}{c} N_{95-95}/N_{\Psi_{\theta }}=\;{\left[\frac{\log\left(1-\Delta \;\theta_{\mathrm{CP}}\right)-\log\left(1-\theta_{\mathrm{CP}}\right)}{\;\log\left(1-\theta_{\mathrm{CP}}\right)\left(1-\Delta \right)\;}\right]}^{2} \end{array}$$

### Determining PYFU

The formulae in the previous sections give the required number of events in each of the active arms. To obtain the required number of events in the counterfactual placebo arm (with equivalent follow-up to the active arms) equations (3), (4), and (6) are divided by $\left(1-\theta_{\mathrm{CP}}\right).$ To obtain the required PYFU per arm (F), we further divide by $\lambda_{P}$ and double this value to obtain the total PYFU. As usual, this value should be adjusted upwards to account for anticipated loss to follow-up.

### Asymptotic assumptions

The formulae above are based on Wald confidence intervals derived via Taylor series expansions and are therefore only valid asymptotically. For the AER based on the counterfactual placebo incidence ( $\Psi_{\lambda }$ ), we previously showed that this approach is reasonably accurate provided there is a minimum of 20 expected events in the hypothetical placebo arm (F $\lambda_{P})$ .^19^ More accurate coverage is obtained by using profile-likelihood confidence intervals for smaller values.^19^ This issue arose for several scenarios that we examined (combinations of high values of $\theta_{\mathrm{CP}}$ and low values of $\Psi_{0}$ ); in these cases, we re-determined the sample size by trial-and-error based on the lower profile-likelihood confidence limit.

## Results

### Sample size for the different estimands

Table 1 shows the required number of events per active arm to achieve 90% power to demonstrate non-inferiority based on the lower 5% confidence limit, according to the estimand, the desired non-inferiority margin ( $\Psi_{0}$ ) and the effectiveness of the control treatment ( $\theta_{\mathrm{CP}}$ ). These sample sizes can be easily scaled for a different level of power or a different lower confidence limit (footnote to Table 1).

**Table 1.** Sample size to demonstrate non-inferiority.

Non-inferiority margin (Δ)	Efficacy of control treatment ( $\theta_{\mathrm{CP}}$ )	Events in each active arm		
		AER based on $\lambda_{P}$	AER based on $\theta_{\mathrm{CP}}$	95-95 method
0.5	0.5	36	105	143
0.5	0.6	16	55	82
0.5	0.7	8	29	48
0.5	0.8	5*	15	27
0.5	0.9	2*	6	13
0.6	0.5	66	152	223
0.6	0.6	30	78	128
0.6	0.7	13	40	74
0.6	0.8	6*	19	42
0.6	0.9	2*	8	21
0.7	0.5	135	249	397
0.7	0.6	60	125	227
0.7	0.7	25	61	132
0.7	0.8	9	28	74
0.7	0.9	3*	11	36
0.8	0.5	344	516	892
0.8	0.6	153	249	511
0.8	0.7	64	117	296
0.8	0.8	22	50	166
0.8	0.9	5*	17	81

Table shows values to achieve 90% power to demonstrate that the lower 5% confidence limit (CL) will exceed the specified non-inferiority margin.

To obtain the required PYFU per arm, values in Table should be multiplied by $1/\lambda_{P}\left(1-\theta_{\mathrm{CP}}\right)$

Values should be scaled by 0.7219 for 80% power and 5% CL; by 1.2270 for 90% power and 2.5% CL; by 0.9165 for 80% power and 2.5% CL.

* Derived from profile-likelihood confidence intervals.

To illustrate how to use Table 1, consider $\theta_{\mathrm{CP}}$ = 80% and Δ = 80%. This gives a required 166 events per active arm using the 95-95 method, 50 events per arm using $\Psi_{\theta }$ , and 22 events per arm using $\Psi_{\lambda }$ . To work out the PYFU required to generate these endpoints, we first multiply these values by 1/(1 − 0.8) = 5 to get the number of required events in a hypothetical placebo arm of the same size (830, 250, and 110 events, respectively). An estimate of λ_P_ is then needed to determine the required PYFU in each trial arm. For example, if λ_P_ = 10/100 PYFU, we would require 8300 PYFU for the 95-95 method, 2500 PYFU for $\Psi_{\theta }$ , and 1100 PYFU for $\Psi_{\lambda }$ . Finally, these values are doubled to get the overall required PYFU.

**Figure 1.**
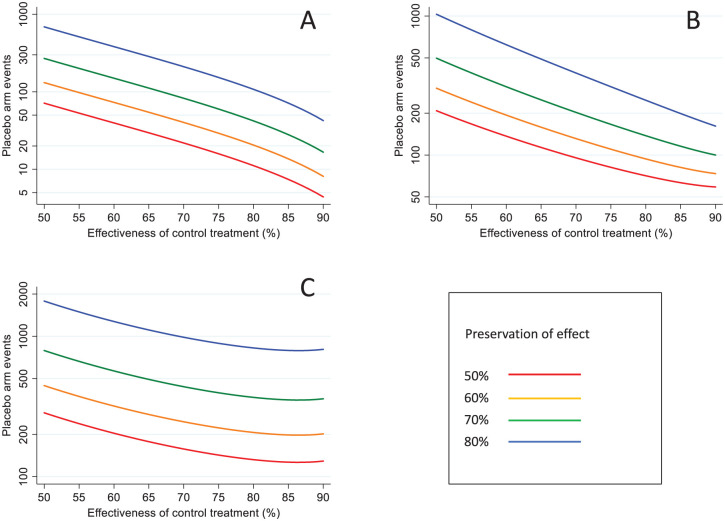
Sample size in terms of the required number of events in the counterfactual placebo group, by non-inferiority margin and effectiveness of the control treatment: (a) AER based on counterfactual placebo incidence, (b) AER based on control treatment effectiveness, (c) 95-95 method. Y-axes plotted on logarithmic scale, using different scales.

**Figure 2.**
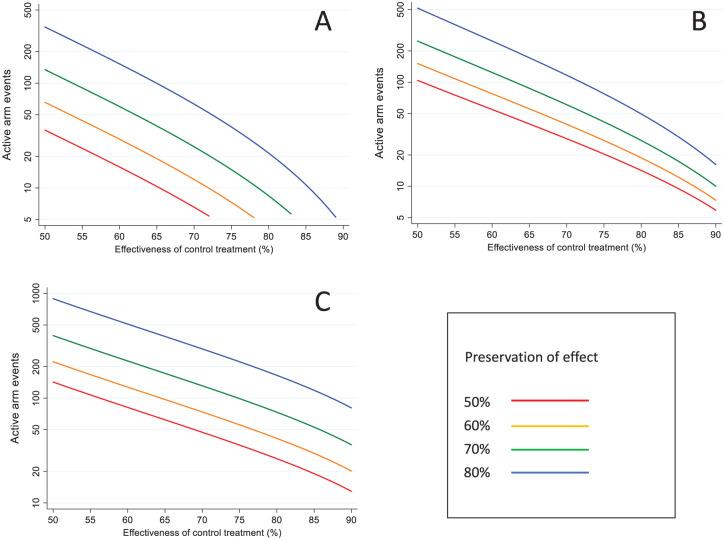
Sample size in terms of the required number of events in each active treatment group, by non-inferiority margin and effectiveness of the control treatment: (a) AER based on counterfactual placebo incidence (b) AER based on control treatment effectiveness (c) 95-95 method. Y-axes plotted on logarithmic scale, using different scales.

The number of required counterfactual placebo endpoints is shown graphically in Figure 1, according to the value of $\Psi_{0}$ . Noting that the y-axes are plotted on a logarithmic scale, the lines in Figure 1(a) and 1(c) are precisely parallel, and those in Figure 1(b) are approximately parallel. For $\Psi_{\lambda }$ and the 95-95 method, increasing Δ from 50% to 60% implies a 1.84-fold increase in the sample size, from 60% to 70% an increase of 2.15-fold, from 70% to 80% an increase of 2.55-fold. Thus, demonstrating non-inferiority at more stringent levels of preservation-of-effect becomes increasingly challenging. Figure 2 similarly shows the number of required endpoints in each of the two active treatment groups.

### Comparison of samples sizes between the different approaches

**Figure 3.**
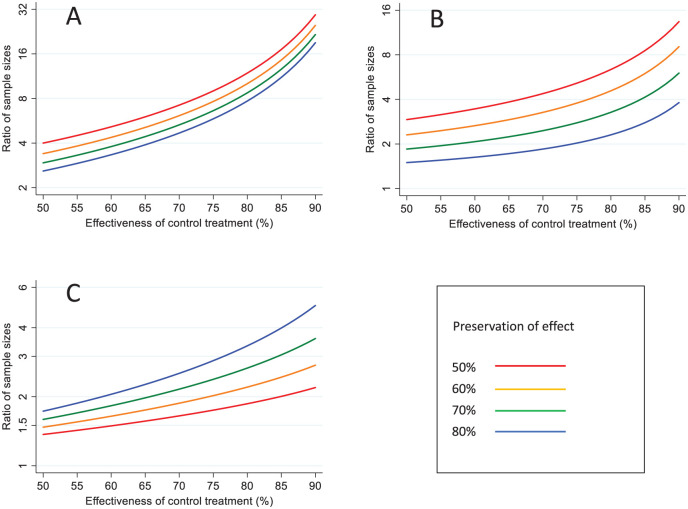
Comparison of sample sizes under different analytical approaches. (a) AER based on counterfactual placebo incidence versus 95-95 method (b) AER based on counterfactual placebo incidence versus AER based control treatment effectiveness (c) AER based on control treatment effectiveness versus 95-95 method. Y-axes plotted on logarithmic scale, using different scales.

The ratio of sample sizes required with the three different approaches is shown in Figure 3. Using $\Psi_{\lambda }$ allows large reductions in sample size, both in comparison with the 95-95 method and $\Psi_{\theta }$ (Figure 3(a) and (b)) and is more advantageous the higher the value of $\theta_{\mathrm{CP}}$ and the lower the value of $\Psi_{0}$ . For example, when $\theta_{\mathrm{CP}}$ = 50%, the 95-95 method requires between a 2.6- and 4.0-fold higher sample size, depending on the value of $\Psi_{0}$ ; when $\theta_{\mathrm{CP}}$ = 80%, it requires between a 7.7- and 11.9-fold higher sample size. Similarly, when $\theta_{\mathrm{CP}}$ = 50%, using $\Psi_{\theta }$ rather than $\Psi_{\lambda }$ requires between a 1.5- and 2.9-fold higher sample size, depending on the value of $\Psi_{0}$ ; when = 80%, it requires between a 2.3- and 6.4-fold higher sample size. To appreciate why smaller sample sizes are needed with $\Psi_{\lambda }$ than with $\Psi_{\theta }$ , note that with the latter we are effectively estimating $\lambda_{P}$ in equation (1) by ${\hat{\lambda }}_{C}/\left(1-\theta_{\mathrm{CP}}\right).$ This has asymptotic variance $\lambda_{C}/\left[F{\left(1-\theta_{\mathrm{CP}}\right)}^{2}\right]$ , which assumes high values as $\theta_{\mathrm{CP}}\to 1$ . The extreme case is a trial with no observed events in either arm. Here, $\Psi_{\lambda }$ is estimable (= 1), and a confidence interval can be derived using likelihood-based methods.^19^ Conversely, $\Psi_{\theta }$ cannot be estimated as ${\hat{\lambda }}_{C}$ = 0, implying ${\hat{\lambda }}_{P}$ = 0 (i.e. the study conveys no information).

If $\lambda_{P}$ is difficult to estimate directly, the estimation of $\Psi_{\theta }$ may be more practicable than $\Psi_{\lambda }$ . The use of $\Psi_{\theta }$ results in a smaller sample size than the 95-95 method, given the same assumption about $\theta_{\mathrm{CP}}$ (Figure 3(c)). This gain is more marked the higher the value of $\theta_{\mathrm{CP}}$ and the higher the value of $\Psi_{0}$ . When $\theta_{\mathrm{CP}}$ = 50%, the 95-95 method requires between a 1.4- and 1.7-fold larger sample size, depending on the value of $\Psi_{0}$ ; when $\theta_{\mathrm{CP}}$ = 80%, it requires between a 2.3- and 6.4-fold larger sample size. The sample size benefit arises from using a different scale to assess non-inferiority.^18^

### Simulation

For all of the scenarios in the Table, we simulated 10,000 trials to quantify the empirical power against the nominal power of 90% (Appendix, Section 5 in the Supplementary Material). For the 95-95 method and $\Psi_{\theta }$ , actual power was generally within ± 1% of the nominal power. For $\Psi_{\lambda },$ the actual power was typically 1%-2% lower than the nominal power. This is due to underestimation of the variance when a simulated trial has more events than expected (Appendix, Section 2 in the Supplementary Material), combined with the right-skewness of the Poisson distribution (from which the observed events are generated).

## Example

DISCOVER was a randomised, double-blind, active-control trial comparing two PrEP regimens in gay men at high risk of HIV infection: TAF/FTC (experimental) versus TDF/FTC (control).^15^ The primary endpoint was an incident HIV infection. Sample size was calculated using the 95-95 method with the following parameters, based on data from three historical placebo-controlled trials of TDF/FTC: lower bound of the 95% CI for the rate ratio = 2.64 (which translates to $\theta_{\mathrm{CP}}=\;1-1/2.64=$ 0.62); preservation-of-effect size (Δ) = 0.5, implying a non-inferiority rate ratio = $\;\sqrt{2.64\;}$ = 1.62; $\;\lambda_{C}=\;\lambda_{E}$ = 1.44/100 PYFU, α = 0.025, β = 0.825. Applying equation (6) gives 72.7 events per arm, requiring 5046 PYFU per arm (the investigators rounded to 72 and 5000). If the trial had instead been analysed using $\;\Psi_{\lambda }$ , with the same parameters, then application of equation (3) shows the trial would have required 13.4 events (931 PYFU) per arm, a 5.4-fold reduction in sample size.

It transpired that the DISCOVER study investigators seriously underestimated the level of treatment adherence and therefore the effectiveness of the PrEP regimens. In the actual trial, only 17 endpoints were observed in total (6 TAF/FTC, 11 TDF/FTC) against the anticipated 144 endpoints. Non-inferiority was formally achieved (rate ratio = 0.55, 95% CI 0.20–1.48), but this finding was highly fragile.^11^ In a *post hoc* calculation, assuming both treatments were 95% effective, we estimated that the actual power of the trial, when analysed by the 95-95 method, was only 14%.

## Discussion

Some scientists may be surprised that using the AER can have such a dramatic effect on sample sizes while remaining a valid estimand. This is due to a fundamental paradox with the rate ratio – as adherence to treatment increases, the number of observed events decreases and the confidence interval for this measure gets increasingly wider.^10^ The AER avoids this paradox, and gives tighter inference the higher the adherence. It is important to note that this advantage does not arise automatically – it comes from making an assumption about one of the counterfactual parameters, and this assumption needs to be approximately correct for valid inference.

Another key finding from our analysis is that much smaller sample sizes are obtained if the AER is estimated via the counterfactual placebo incidence rate rather than the counterfactual treatment effectiveness, particularly when effectiveness is high. In HIV prevention research, the critical importance of estimating the background incidence rate is now widely recognised, and various ways of achieving this have been proposed.^20^ In other contexts, depending on the epidemiology of the disease and the availability of surveillance systems, estimating the background event rate may not be feasible, and using the counterfactual treatment effectiveness may be the only possible approach.^12^

### Specifying the sample size parameters

We address a possible point of confusion when using the AER. In the analysis, estimation of the AER is performed via *either* the counterfactual treatment effectiveness *or* the counterfactual placebo incidence. However, at the design stage, estimation of the sample size requires specification of *both* parameters, regardless of the analytical approach. Both counterfactual parameters will usually be subject to considerable uncertainty, and to avoid an under-powered study, it is prudent to use conservatively low values. If using the counterfactual treatment effectiveness, one can adopt the same approach as with the 95-95 method. We note that the term ‘constancy’ assumption is somewhat misleading – the actual effectiveness in the trial must be equal to *or greater than* this value for valid inference about non-inferiority. Also, between planning the trial and its completion, further information may emerge, and there is no objection in principle to using updated estimates in the final analysis provided these are carefully justified. Our sample size formulae involve single, fixed values for $\lambda_{P}\;\mathrm{and}\;\theta_{\mathrm{CP}}$ . An interesting alternative approach would be to specify prior distributions for these parameters under a Bayesian framework, and to determine sample size via predictive power.^21^ Finally, although demonstrating 50% preservation-of-effect has become the *de facto* standard in many areas of research, other researchers have suggested the use of more stringent levels, particularly in the case of highly effective treatments.^5,6^

## Supplemental Material

sj-docx-1-ctj-10.1177_17407745251377435 – Supplemental material for Sample size estimation for the averted events ratio Supplemental material, sj-docx-1-ctj-10.1177_17407745251377435 for Sample size estimation for the averted events ratio by David T Dunn, Oliver T Stirrup and David V Glidden in Clinical Trials
