# Supplementary material for: Sample size estimation for the averted events ratio
Source: Clin Trials. 2025 Oct 23;23(1):5–12. doi: 10.1177/17407745251377435 (PMC12614357; doi:10.1177/17407745251377435)
Supplement: sj-docx-1-ctj-10.1177_17407745251377435 – Supplemental material for Sample size estimation for the averted events ratio [file sj-docx-1-ctj-10.1177_17407745251377435.docx]

**APPENDIX**

**1. Notation**

Denote the counterfactual placebo, control, and experimental arms by the subscripts P, C, and E, respectively. Let λ (subscripted by P, C, or E) represent the relevant incidence rate. Assume that we observe equal person-years follow-up in the control and experimental arms (F). Let X_C_ and X_E_ be random variables denoting the number of observed events, where $X_{C}\text{\textasciitilde Poi}\left( F\text{λ}_{\text{C}} \right)$ and$X_{\text{E}}\text{\textasciitilde Poi}\left( F\text{λ}_{\text{E}} \right)$. The (unobserved) effectiveness of the control treatment relative to placebo is $\theta_{\mathrm{CP}}=1-{\lambda_{C}}/{\lambda_{P}}$.

**2. AER as a function of the counterfactual placebo incidence rate (**$\boldsymbol{\Psi}_{\boldsymbol{\lambda}}$**)**

The main formulation of the averted events ratio (AER) is

| $\Psi_{\lambda}= \frac{\lambda_{P}- \lambda_{E}}{\lambda_{P}- \lambda_{C}}$ |  |
| --- | --- |

Based on a first-order Taylor series expansion,

$$\log\Psi_{\lambda}=\log\left( \lambda_{P}- \lambda_{E} \right)- \log\left( \lambda_{P}- \lambda_{C} \right)$$

Regarding λ_P_ as fixed and constant,

$$\mathrm{var}\left( \log\hat{\Psi}_{\lambda} \right) \to\frac{\mathrm{var}\left( \hat{\lambda}_{E} \right)}{\left( \lambda_{P}-\hat{\lambda}_{E} \right)^{2}}+\frac{\mathrm{var}\left( \hat{\lambda}_{C} \right)}{\left( \lambda_{P}-\hat{\lambda}_{C} \right)^{2}}$$

The RHS is estimated by

$$\frac{{\hat{\lambda}_{E}}/F}{\left( \lambda_{P}-\hat{\lambda}_{E} \right)^{2}}+\frac{{\hat{\lambda}_{C}}/F}{\left( \lambda_{P}-\hat{\lambda}_{C} \right)^{2}}= \frac{X_{E}}{\left( F\lambda_{P}-X_{E} \right)^{2}}+\frac{X_{C}}{\left( F\lambda_{P}-X_{C} \right)^{2}}$$

If the experimental and control agents are equally effective (${i.e. \theta}_{\mathrm{EP}}=\theta_{\mathrm{CP}}$), both $X_{E}\mathrm{and}X_{C}\to$

$F\lambda_{P} \left( 1- \theta_{\mathrm{CP}} \right)$, and therefore

$$\mathrm{var}\left( \log\hat{\Psi}_{\lambda} \right)\to\frac{2\left( 1-\theta_{\mathrm{CP}} \right)}{F{\lambda_{P} \theta_{\mathrm{CP}}}^{2}}$$

Non-inferiority is demonstrated if the **lower** (1-$\alpha)$ confidence limit for $\Psi_{\lambda}$ exceeds the pre-specified non-inferiority margin, Δ. This implies

$$\log\hat{\Psi}_{\lambda}- \Phi^{-1}\left( 1-\alpha\right) \sqrt{\mathrm{var}\left( \log\hat{\Psi}_{\lambda} \right)}>\log\Delta$$

As${the assumption \theta}_{\mathrm{EP}}=\theta_{\mathrm{CP}}$ implies $E\left( \log\hat{\Psi}_{\lambda} \right)=0$, the probability of this outcome is

$$= 1- \Phi\left\{ \frac{\sqrt{F\lambda_{P}} \theta_{\mathrm{CP}} \log\Delta}{\sqrt{2 \left( 1-\theta_{\mathrm{CP}} \right)}}+\Phi^{-1}\left( 1-\alpha\right) \right\}$$

Letting $\beta$ denote the probability of declaring non-inferiority (power), the required number of expected events in the counterfactual placebo arm,

$$\begin{aligned} F\lambda_{P}= \frac{2 Z\left( \alpha,\beta\right) \left( 1-\theta_{\mathrm{CP}} \right)}{\left( \theta_{\mathrm{CP}} \log\Delta\right)^{2}} \#(1) \end{aligned}$$

where $Z\left( \alpha,\beta\right)= \left[ \Phi^{-1}\left( 1-\alpha\right)+ \Phi^{-1}\left( \beta\right) \right]^{2}$

To obtain the required number of expected events in each of the active treatment arms, equation (1) is scaled by a factor $\left( 1-\theta_{\mathrm{CP}} \right)$ to yield

$$\begin{aligned} 2 Z\left( \alpha,\beta\right) \left[ \frac{\left( 1-\theta_{\mathrm{CP}} \right)}{\theta_{\mathrm{CP}}\log\Delta} \right]^{2} \end{aligned}$$

**3. AER as a function of the effectiveness of the control treatment (**$\boldsymbol{\Psi}_{\boldsymbol{\theta}}$**)**

In this formulation

$$\begin{aligned} \Psi_{\theta}= \frac{1-{\lambda_{E}}/{\lambda_{C}}\left( 1-\theta_{\mathrm{CP}} \right)}{\theta_{\mathrm{CP}}} \#(2) \end{aligned}$$

Note that $\Psi_{\theta}$is a linear function of the rate ratio, ${\lambda_{E}}/{\lambda_{C}}$ , and that the upper CL for ${\lambda_{E}}/{\lambda_{C}}$ corresponds to the lower CL for $\Psi_{\theta}.$ Rearranging equation (2),

$$\log\left( {\lambda_{E}}/{\lambda_{C}} \right)=\log\left( \frac{1-\Psi_{\theta} \theta_{\mathrm{CP}}}{1-\theta_{\mathrm{CP}}} \right)$$

Non-inferiority is demonstrated if the **upper** (1-$\alpha)$ confidence limit for $\log\left( {\lambda_{E}}/{\lambda_{C}} \right)$is less than

$$\log\left( \frac{1-\Delta\theta_{\mathrm{CP}}}{1-\theta_{\mathrm{CP}}} \right)$$

$$\mathrm{var}\left[ \log\left( {\lambda_{E}}/{\lambda_{C}} \right) \right] is estimated by \left( \frac{1}{X_{E}}+ \frac{1}{X_{C}} \right), \mathrm{which}\to\frac{2}{F\lambda_{P}\left( 1-\theta_{\mathrm{CP}} \right)}$$

Again assuming λ_E_ = λ_C ,_ the probability of declaring non-inferiority

$$= 1- \Phi\left\{ -\log\left( \frac{1-\Delta\theta_{\mathrm{CP}}}{1-\theta_{\mathrm{CP}}} \right)\sqrt{\frac{F\lambda_{P}\left( 1-\theta_{\mathrm{CP}} \right)}{2}}+\Phi^{-1}\left( 1-\alpha\right) \right\}$$

To achieve a pre-specified power $\beta$, the required number of expected events in the counterfactual placebo arm,

$$F\lambda_{P}= \frac{2 Z\left( \alpha,\beta\right)}{{\left( 1-\theta_{\mathrm{CP}} \right) \left[ \log\left( 1-{\Delta\theta}_{\mathrm{CP}} \right)-\log\left( 1-\theta_{\mathrm{CP}} \right) \right]}^{2}}$$

Again, scaling by a factor $\left( 1-\theta_{\mathrm{CP}} \right),$the required number of expected events in each of the active treatment arms is

$$\frac{2 Z\left( \alpha,\beta\right)}{\left[ \log\left( 1-{\Delta\theta}_{\mathrm{CP}} \right)-\log\left( 1-\theta_{\mathrm{CP}} \right) \right]^{2}}$$

**4. 95-95 Method**

Under the 95-95 method, preservation is measured by ${{\phi=\beta}_{\mathrm{PE}}}/{\beta_{\mathrm{PC}}}$, where $\beta_{\mathrm{PE}}=\log\left( {\lambda_{P}}/{\lambda_{E}} \right)$ and $\beta_{\mathrm{PC}}=\log\left( {\lambda_{P}}/{\lambda_{C}} \right)$. We wish to demonstrate that $\phi$ > $\Delta$ $\Rightarrow\beta_{\mathrm{PE}}$ > $\Delta$ $\beta_{\mathrm{PC}}$. As $\beta_{\mathrm{PE}}$is not observed, we invoke the “constancy” assumption, $\beta_{\mathrm{PE}}= \beta_{\mathrm{PC}}+ \beta_{\mathrm{CE}}$. $\beta_{\mathrm{CE}}$ is directly observed in the active-control trial, and $\beta_{\mathrm{PC}}$is imputed from the results of previous placebo-controlled trials. Thus

$\phi$ > $\Delta$ $\Rightarrow\beta_{\mathrm{CE}}$ > $\beta_{\mathrm{PC}} \left( \Delta-1 \right) \Rightarrow\beta_{\mathrm{EC}}$ < ${-\beta}_{\mathrm{PC}} \left( \Delta-1 \right)$= $-\log\left( 1-\theta_{\mathrm{CP}} \right)\left( 1- \Delta\right)$

Non-inferiority is therefore demonstrated if the **upper** (1-$\alpha)$ confidence limit for $\beta_{\mathrm{EC}}$ is less than $-\log\left( 1-\theta_{\mathrm{CP}} \right)\left( 1- \Delta\right)$

By analogy with Section 3, the probability of declaring non-inferiority

$$= 1- \Phi\left\{ - log \left( 1-\theta_{\mathrm{CP}} \right)\left( 1-\Delta\right)\sqrt{\frac{F\lambda_{P}\left( 1-\theta_{\mathrm{CP}} \right)}{2}}+\Phi^{-1}\left( 1-\alpha\right) \right\}$$

and the required number of expected events in the counterfactual placebo arm to achieve power $\beta$,

$$F\lambda_{P}= \frac{2 Z \left( \alpha,\beta\right)}{{\left( 1-\theta_{\mathrm{CP}} \right)\left[ \log\left( 1-\theta_{\mathrm{CP}} \right)\left( 1- \Delta\right) \right]}^{2}}$$

Scaling by a factor $\left( 1-\theta_{\mathrm{CP}} \right),$the required number of expected events in each of the active treatment arms is

$$\frac{2 F\left( \alpha,\beta\right)}{\left[ \log\left( 1-\theta_{\mathrm{CP}} \right)\left( 1-\Delta\right) \right]^{2}}$$

This accords with the expressions derived by Fleming (Stat Med 2008, 27:317-322) and Schoenfeld (Biometrics 1983, 39:499-503).

**5. Simulations to verify accuracy of sample size formulae**

10,000 trials were simulated for each of the rows in Table 1, and the empirical power calculated. Ideally, the empirical power should equal 90%.

| **Non-inferiority margin (Δ)** | **Efficacy of control treatment (**$\boldsymbol{\theta}_{\mathbf{CP}}$**)** | **Empirical power (nominal=0.9000)** | | |
| --- | --- | --- | --- | --- |
|  |  | **AER based on** $\boldsymbol{\lambda}_{\mathbf{P}}$ | **AER based on** $\boldsymbol{\theta}_{\mathbf{CP}}$ | **95-95 method** |
| 0.5 | 0.5 | 0.8942 | 0.8971 | 0.9038 |
| 0.5 | 0.6 | 0.8987 | 0.8937 | 0.8984 |
| 0.5 | 0.7 | 0.8631 | 0.8900 | 0.9015 |
| 0.5 | 0.8 | 0.9013* | 0.8903 | 0.8861 |
| 0.5 | 0.9 | 0.8889* | 0.8614 | 0.8980 |
| 0.6 | 0.5 | 0.8950 | 0.8971 | 0.9041 |
| 0.6 | 0.6 | 0.8813 | 0.8922 | 0.8976 |
| 0.6 | 0.7 | 0.8747 | 0.8982 | 0.9024 |
| 0.6 | 0.8 | 0.8784* | 0.8824 | 0.8936 |
| 0.6 | 0.9 | 0.9172* | 0.8805 | 0.8940 |
| 0.7 | 0.5 | 0.8981 | 0.8991 | 0.8980 |
| 0.7 | 0.6 | 0.8973 | 0.8983 | 0.8981 |
| 0.7 | 0.7 | 0.8926 | 0.8930 | 0.8975 |
| 0.7 | 0.8 | 0.8884 | 0.8906 | 0.8981 |
| 0.7 | 0.9 | 0.9223* | 0.8916 | 0.8962 |
| 0.8 | 0.5 | 0.8975 | 0.9021 | 0.9017 |
| 0.8 | 0.6 | 0.8958 | 0.9001 | 0.9026 |
| 0.8 | 0.7 | 0.8931 | 0.9002 | 0.8961 |
| 0.8 | 0.8 | 0.8898 | 0.8961 | 0.8996 |
| 0.8 | 0.9 | 0.8700* | 0.9012 | 0.8938 |

* Using profile-likelihood confidence interval

**6. Alternative models**

Our analysis has been based on a Poisson model, which assumes time-constant incidence in each of the groups. Alternative approaches are available if there is evidence that this assumption is violated. Using $\Psi_{\theta}$, one can fit a Cox proportional hazards model, substituting the estimated hazard ratio for ${\lambda_{E}}/{\lambda_{C}}$ in equation (2). Using $\Psi_{\lambda}$ , the analysis could proceed in terms of the estimated survival functions at the end of follow-up, $S\left( \tau\right)=\int_{0}^{\tau} \lambda\left( u \right)\mathrm{du}$. This is equivalent to a binary model if there is no loss to follow-up, in which case the AER would be estimated by $\Psi_{\pi}= \left( \pi_{P}- \pi_{E} \right)/\left( \pi_{P}- \pi_{C} \right)$. Sample size formula for this case are straightforward to develop, although the number of events per active arm includes a term for $\pi_{P}.$

**7. STATA code**

* AER_sample_size.do

* gives number of events per arm

version 15.1

scalar drop _all

// specify each of the following parameters

scalar power=0.9 // desired power

scalar alpha=0.05 // NI inference based on lower (1-alpha) CL of estimand

scalar delta=0.8 // preservation-of-effect size

scalar theta=0.8 // treatment effectiveness (assumed equal for two active treatments)

scalar lamda_p=0.1 // counterfactual placebo incidence rate

scalar Z=(invnorm(power)+invnorm(1-alpha))^2

// AER based on placebo incidence

scalar placebo_events_lamda=ceil((2*Z*(1-theta))/(theta*log(delta))^2) // rounding up

scalar active_events_lamda=ceil(placebo_events_lamda*(1-theta))

scalar FU_lamda=active_events_lamda/lamda_p

// AER based on treatment effectiveness

scalar placebo_events_theta= ceil(Z*(2/(1-theta))* (log((1-delta*theta)/(1-theta)))^(-2))

scalar active_events_theta =ceil(placebo_events_theta*(1-theta))

scalar FU_theta=active_events_theta/lamda_p

// 95-95 method

scalar active_events_9595= ceil(2*Z*(log(1-theta)*(1-delta))^(-2))

scalar FU_9595=active_events_9595/lamda_p

scalar list
